# Neurobiological pathways underlying brain tumor progression: basis for oncogenicity and opportunities for immunotherapeutic intervention

**DOI:** 10.3389/fonc.2026.1763401

**Published:** 2026-03-19

**Authors:** Carrie E. Andrews, Jenny Zilberberg, Raul Perez-Olle, David Andrews, Mark A. Exley

**Affiliations:** 1 Department of Neurological Surgery, Thomas Jefferson University, Philadelphia, PA, United States; 2 Imvax, Inc., Philadelphia, PA, United States; 3 Biodemak LLC, Hoboken, NJ, United States; 4 Department of Life Sciences, Imperial College, London, United Kingdom

**Keywords:** brain metastases, cancer stem cells, glioblastoma, neuron, neuronal, tumor

## Abstract

Both primary and metastatic brain tumors rely on signals from the surrounding environment for their survival and progression. In particular, the most common and lethal brain cancer, glioblastoma (GBM), derived from glial cells (astrocytes or microglia), has been shown to integrate into synaptic networks and to receive paracrine signals from neighbouring tumor microenvironment (TME) cells. There is increasing evidence that metastatic disease in the brain exhibits similar behavior. The TME both maintains malignant cells and is maintained by them, a process that relies on cancer stem cells (CSCs). These stem cells and their signaling mechanisms, including in the case of GBM, “GSCs,” provide possible novel targets for immunotherapy. In this review, we will discuss the integration of primary and malignant brain tumors into normal synaptic networks, the role of tumor stem cells and the TME in this integration, and the potential for immunotherapeutic targeting of these processes.

## Introduction

High grade gliomas (HGGs) are fast-growing, highly aggressive brain tumors with devastating prognosis that arise from glial cells or their precursors (1). They correspond to WHO grade III and Grade IV tumors in the World Health Organization (WHO) classification system and are defined by both their histological as well as molecular features (2). Glioblastoma (GBM), the most common and fatal primary brain tumor in adults, continues to have a median overall survival of 15–18 months (3). Therapies remain limited, although clinical studies continue, including vaccine-type immunotherapies, with varied results (4–6).

Brain metastases remain the most common form of brain tumor and affect one in five patients with systemic cancers (7). Despite improved prognosis overall in patients with somatic malignancies, prognosis in those with disease metastatic to the brain also remains poor. Furthermore, metastases incur significant morbidity from a neurologic standpoint (8). There has been increasing concern that the subpopulation of tumor cells that make up brain metastases differs biologically from their somatic tumors of origin, highlighting a need for alternative therapies directed specifically at tumors in the central nervous system (9).

## Impact of neuronal signaling on tumorigenesis and proliferation in primary and metastatic brain tumors

**Figure 1:**
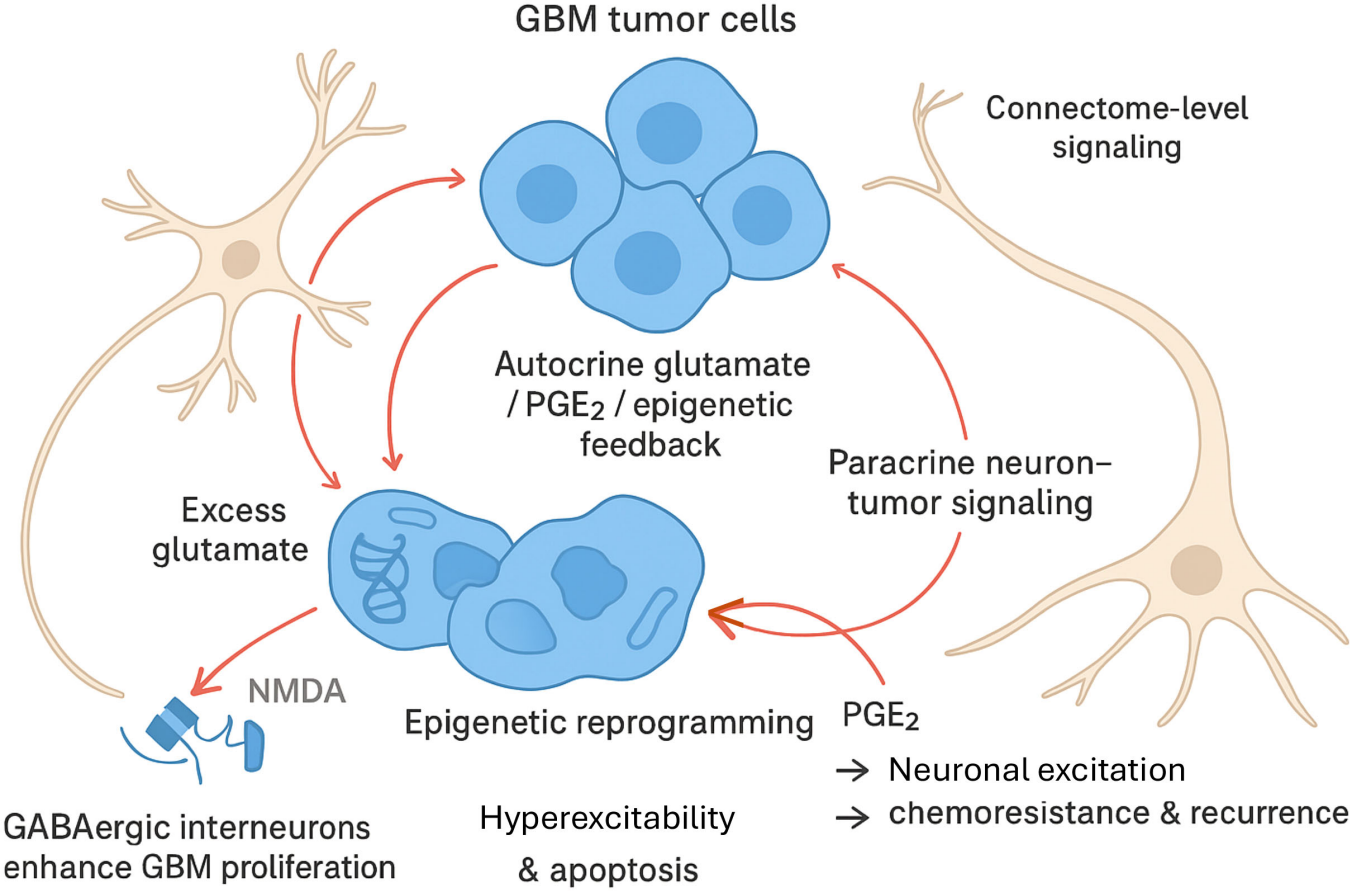
Neuronal autocrine and paracrine signals modulate tumorigenesis of non-neuronal-derived brain tumors. Neuronal signaling plays a critical role in driving glioblastoma (GBM) tumorigenesis, proliferation, and treatment resistance, both locally within the tumor microenvironment and across broader neural networks. GBM cells release excess glutamate, activating NMDA and AMPA receptors on surrounding neurons (shown with axons), promoting tumor growth and invasion. GBM cells also form direct glutamatergic synapses with neurons, while GABAergic interneurons have been shown to further enhance GBM proliferation. NMDA, N-methyl-D-aspartate; AMPA, α-amino-3-hydroxy-5-methyl-4-isoxazolepropionic acid.

Neuronal signaling modulates the tumorigenesis and proliferation of non-neuronal-derived brain tumors on both a localized level, via autocrine and paracrine mechanisms, as well as on a more global scale throughout the “connectome” (**Figure 1**). At the local level, glutamate has long been implicated in pathogenesis of GBM. GBM cells release excess glutamate into the tumor microenvironment, which activates ionotropic N-methyl-D-aspartate (NMDA) and α-amino-3-hydroxy-5-methyl-4-isoxazolepropionic acid (AMPA) receptors of normal surrounding neurons. This results in glutamate excitotoxicity, a combination of hyperexcitability and apoptosis of these neurons, which contributes to the proliferation and invasion of GBM cells (10). Further, direct glutamatergic synapses between GBM cells and normal neurons, mediated by glutamate, drive tumor growth and invasion (11). More recently, gamma-aminobutyric acid (GABA)-ergic interneurons have been shown to drive GBM proliferation *in vivo* (12). Neuron-tumor interactions also drive chemoresistance and tumor recurrence via prostaglandin E2-induced neuronal excitation (13). Epigenetic reprogramming modifies the downstream effects of neurotransmitters within glioma cells, fueling their integration into neural networks (14).

Integration of GBM into neural networks is another driver of its progression and treatment resistance (**Figure 1**) (15). Proteomic analysis of recurrent GBM has demonstrated that activation of the Wnt/Planar Cell Polarity (Wnt/PCP) signaling pathway and B-Raf proto-oncogene, serine/threonine (BRAF) protein kinase modulates a neuronal transition of tumor cells, allowing recurrent GBM cells to integrate into neural tissue (16). Ultralong membrane protrusions in astrocytomas create a network that allows communication between tumor cells over long distances via microtubule-associated gap junctions, which allows for brain invasion over long distances (17). These projections contribute to resistance to surgical and radiosurgical intervention (18). Human GBM organoids transplanted into adult mice showed rapid development of extensive interactions between GBM cells and both cortical and subcortical structures bilaterally (19). This integration into the brain milieu likely contributes to treatment resistance (20). Further, the dysregulation of normal synaptic connections in higher cortical structures, as manifested by hyperexcitability, likely contributes to clinical manifestations of GBM, such as cognitive dysfunction and seizures (21).

Although less is known about neuronal integration and signaling in the case of brain metastases, this has increasingly been a field of inquiry, particularly given the prevalence and poor prognosis of such tumors (22). Small-cell lung cancer (SCLC) metastases have been found to be affected by both paracrine and synaptic signaling, similarly to GBM cells, mediated by GABA and glutamate. In a cell culture experiment by Savchuk and colleagues, there was significant upregulation of proliferation in SCLC cells cultured with neuronal cells, as compared to SCLC monoculture (23). In addition to such paracrine signaling, models of basal-type breast cancer have demonstrated the ability to form pseudo-tripartate glutamatergic synapses to drive brain metastasis (24). SCLC cells obtained from biopsies of human brain metastases were found to have a unique synaptic signature on RNA sequencing, as compared to somatic site metastases. Further, electron microscopy of allografts and xenografts of SCLC metastases revealed synaptic connections between SCLC cells and neurons, as well as pseudo-tripartate synapses similar to those seen in brain cancer metastases. These SCLC cells had synaptic currents, and optogenetic models suggest that the neuronal signaling activity allowed by such synaptic activity drives tumor progression, as in GBM (23).

## Cancer stem cells’ role and the tumor microenvironment in GBM and metastatic brain tumors

GSCs are a self-renewing component of these tumors that play a key role in their continued growth and invasion. They rely on a symbiotic relationship with the TME, which both facilitates the functions of and reciprocally depends on GSCs for its maintenance (25). Recent studies suggest that the brain’s neuronal connections can directly support, expand, and reprogram GSCs. For example, DOC-2/DAB2 interactive protein (DAB2IP) suppresses GSC properties by targeting the synaptic proteins neuroligin 3 (NLGN3), which promotes glioma proliferation through the PI3K-mTOR pathway (26). Venkatesh et al. showed that patient-derived orthotopic xenografts of pediatric GBM, Diffuse Intrinsic Pontine Glioma (DIPG) and adult GBM fail to grow in *Nlgn3* knockout mice. Furthermore, ADAM10 inhibitors capable of cleaving NLGN3 prevent its release into the tumor microenvironment and significantly blocked HGG xenograft growth (27).

Interestingly, GBM regions with heightened neuronal connectivity exhibit localized immunosuppression, characterized by distinct immune cell compositions and an enrichment of immunosuppressive tumor-associated macrophages (TAMs). As a major cellular component of the TME, these pro-tumor TAMs not only promote tumor growth and invasion but also suppress immune-mediated anti-tumor responses (14). Nejo et al., recently found that knockout of Thrombospondin-1 (TSP1/*Thbs1*) in GBM cells suppressed synaptogenesis and glutamatergic neuronal hyperexcitability. Importantly, TSP1 knockout restored antigen presentation-related genes promoting the infiltration of pro-inflammatory TAMs and CD8 + T cells in the tumor and alleviated TAM-mediated T cell suppression (28).

Mutations in key GBM-associated genes constitute another mechanism that can simultaneously shape a TME that supports cancer growth while enhancing GSC stemness and survival. For example, mutations in arsenite-resistance protein 2 (ARS2), a zinc finger protein, have been shown to promote both the stem-like properties of GSCs and the immunosuppressive M2-like polarization of TAMs, two complementary mechanisms that facilitate tumor persistence and proliferation (15). CXCL-8 (also known as IL-8) is a chemokine expressed at higher rates in some GBM phenotypes and is associated with shorter survival (29). Its expression by GSCs in GBM drives intrinsic survival and proliferation of the GSCs themselves via PI3K/AKT and NF-kB signaling. Further, it contributes to the maintenance of the TME via the CXCR2-JAK2/STAT3 pathway in TAMs, a paracrine mechanism by which TAMs are differentiated to an M2 phenotype (30).

STAT3 is a key transcription factor involved in the regulation of multiple stem cell types, including GCS (31). The interleukin-6 (IL-6)/STAT3 signaling axis is well established as a driver of cancer progression across diverse tumor types, including GBM. Using murine models and clinical samples, Lamano et al. demonstrated that GBM-derived IL-6 is both necessary and sufficient to induce myeloid PD-L1 expression through a STAT3-dependent mechanism (32). Furthermore, IL-6 signaling promotes GSC survival via STAT3 activation, and disruption of this pathway, either through STAT3 inhibitors or by blocking IL-6/IL-6Rα, triggers GSC apoptosis and prolongs survival in mouse models of intracranial human glioma (33).

Brain metastases are derived from circulating tumor cells (CTCs), nests of cells derived from a somatic tumor that have been found to have properties of increased stemness (34). The TME of brain metastases is different from the TME of their somatic site of origin, owing in part to the presence of the blood brain barrier (BBB) (35). Rather than through a random process, CTCs from cancers of different somatic origins, e.g. breast, lung, and melanoma, express alpha-2,6, sialyltransferase, an adhesion molecule that allows for central nervous system penetration (36). As compared to the cells at the site of origin, the unique properties of these CTCs that allow for survival within the brain microenvironment confer some extent of treatment resistance (35). Identification of unique characteristics within the TME of brain metastases may, however, allow for precision targeting of these tumors for a personalized therapeutic approach (37).

## Brain cancer and specific GBM immunotherapies and targeting of GSCs

Immunotherapy is now considered the fourth pillar of cancer treatment, along with surgery, chemotherapy, and radiation therapy, and its potential in treatment of brain cancers is now beginning to be realized (38, 39). GSCs are particularly resistant to conventional chemo- and radiation therapy, which may even sustain or contribute to stemness in cancer cell populations (40). Given the role of CSCs in tumor survival and metastasis, however, they are a natural and necessary target for therapeutic intervention (41). Therapeutics such as molecules, antibodies, vaccines, and chimeric antigen receptor (CAR)-T cells have all been utilized in the targeting of the multiple intracellular and extracellular processes that propagate and sustain CSCs (41). CSCs do commonly share biomarkers, and such biomarkers provide potential targets for immunotherapies (42).

GSC-derived tumor-associated antigens (TAAs) have been the focus of several therapeutic strategies aimed at eradicating this self-renewing cell population responsible for tumor initiation, progression, recurrence, and resistance to treatment. Finocchiaro et al. conducted a phase I clinical trial with ICT-107, a dendritic cell (DC) vaccine targeting six different TAAs: HER2, TRP-2, gp100, MAGE-1, IL13Rα2, and AIM-2. In this study, DCs were pulsed with class I (HLA-A1 and HLA-A2)-restricted peptides derived from these proteins (43). HER2, TRP-2, AIM-2, and IL13Rα2 are considered GSC-associated antigens, as previously demonstrated in the preclinical rat 9L gliosarcoma model (44) and evaluated in positive a Phase 1 and subsequent Phase 2 randomized trial reporting 2 month improvements in median progression-free survival and overall survival (45, 46).

General CSC antigens targeted through CAR-T cell therapy include B7-H3, EGFRvIII, and GRP78. NKG2D ligands, which are upregulated in GSCs, have proven to be an effective target. Second-generation anti-NKG2D CAR T-cells have been shown to be able to eliminate GBM cells and GSCs (47). It is well established that each patient’s tumor contains a diverse population of cells, and that the antigens expressed by GSCs can differ from those on other tumor cells, making it difficult to develop mono-targeted treatments that effectively and universally target all cancer stem cells (38, 39). In this regard, the role of Semaphorin 3A in EGFRvIII function suggests a novel vulnerability (48).

Another strategy involves IGV-001, which is currently being evaluated in a Phase 2b randomized clinical trial. IGV-001, the first therapeutic candidate developed from the Goldspire^®^ platform (5, 49, 50), leverages the bulk of resected GBM tissue as its source of tumor antigens. By being antigen inclusive, this approach has the potential to generate a diverse repertoire of anti-tumor T cells capable of targeting many often unknown and patient-specific GBM antigens. These can include GSC-derived antigens, due to Goldspire^®^’s broad, antigen-agnostic, whole tumor-derived manufacturing platform (5, 49, 50). This approach also has potential with brain metastatic disease, since it targets truncal antigens present in the primary tumor, even if not targeting new antigens generally expressed in metastases (51).

## Summary

It is becoming increasingly clear that neurobiological processes, such as neuron-tumor interactions, neurotransmitter signaling, and neural circuit remodeling, are closely linked to the growth and progression of malignant brain tumors. In this review, we discussed recent findings that underscore the role of neuro-signaling mechanisms in shaping GCSs and the TME, and highlight emerging therapeutic strategies aimed at targeting this self-renewing tumor population.

Other strategies to target brain cancers, including primary and metastatic disease, may work in concert with the neuron-tumor approaches discussed above. For example, multiple approaches are being employed to target myeloid suppression in all cancers, including those that begin or end up in the brain (39, 52). Further research may elucidate how neurobiological interactions generate immunologically relevant vulnerabilities, thereby directly or indirectly highlighting neuro-signaling networks as promising targets for next-generation immunotherapies, alone and in combination.

## References

[1] Zhang X Zhang W Mao XG Cao WD Zhen HN Hu SJ . Malignant intracranial high grade glioma and current treatment strategy. Curr Cancer Drug Targets. (2019) 19:101–8. doi: 10.2174/1568009618666180530090922. PMID: 29848277

[2] Louis DN Perry A Wesseling P Brat DJ Cree IA Figarella-Branger D . The 2021 WHO classification of tumors of the central nervous system: a summary. Neuro Oncol. (2021) 23:1231–51. doi: 10.1093/neuonc/noab106. PMID: 34185076 PMC8328013

[3] Wen PY Weller M Lee EQ Alexander BM Barnholtz-Sloan JS Barthel FP . Glioblastoma in adults: a Society for Neuro-Oncology (SNO) and European Society of Neuro-Oncology (EANO) consensus review on current management and future directions. Neuro Oncol. (2020) 22:1073–113. doi: 10.1093/neuonc/noaa106. PMID: 32328653 PMC7594557

[4] Liau LM Ashkan K Brem S Campian JL Trusheim JE Iwamoto FM . Association of autologous tumor lysate-loaded dendritic cell vaccination with extension of survival among patients with newly diagnosed and recurrent glioblastoma: a phase 3 prospective externally controlled cohort trial. JAMA Oncol. (2023) 9:112–21. doi: 10.1001/jamaoncol.2022.5370. PMID: 36394838 PMC9673026

[5] Andrews CE Zilberberg J Perez-Olle R Exley MA Andrews DW . Targeted immunotherapy for glioblastoma involving whole tumor-derived autologous cells in the upfront setting after craniotomy. J Neuro Oncol. (2023) 165:389–98. doi: 10.1007/s11060-023-04491-4. PMID: 38017340 PMC10942892

[6] Bloch O Lim M Sughrue ME Komotar RJ Abrahams JM O’Rourke DM . Autologous heat shock protein peptide vaccination for newly diagnosed glioblastoma: impact of peripheral PD-L1 expression on response to therapy. Clin Cancer Res. (2017) 23:3575–84. doi: 10.1158/1078-0432.CCR-16-1369. PMID: 28193626 PMC5511566

[7] Sacks P Rahman M . Epidemiology of brain metastases. Neurosurg Clin N Am. (2020) 31:481–8. doi: 10.1016/j.nec.2020.06.001. PMID: 32921345

[8] Achrol AS Rennert RC Anders C Soffietti R Ahluwalia MS Nayak L . Brain metastases. Nat Rev Dis Primers. (2019) 5:5. doi: 10.1038/s41572-018-0055-y. PMID: 30655533

[9] Valiente M Ahluwalia MS Boire A Brastianos PK Goldberg SB Lee EQ . The evolving landscape of brain metastasis. Trends Cancer. (2018) 4:176–96. doi: 10.1016/j.trecan.2018.01.003. PMID: 29506669 PMC6602095

[10] Moriarty C Gupta N Bhattacharya D . Role of glutamate excitotoxicity in glioblastoma growth and its implications in treatment. Cell Biol Int. (2025) 49:421–34. doi: 10.1002/cbin.70005. PMID: 40014265 PMC11994879

[11] Venkataramani V Tanev DI Strahle C Studier-Fischer A Fankhauser L Kessler T . Glutamatergic synaptic input to glioma cells drives brain tumour progression. Nature. (2019) 573:532–8. doi: 10.1038/s41586-019-1564-x. PMID: 31534219

[12] Barron T Yalçın B Su M Byun YG Gavish A Shamardani K . GABAergic neuron-to-glioma synapses in diffuse midline gliomas. Nature. (2025) 639:1060–8. doi: 10.1038/s41586-024-08579-3. PMID: 39972132 PMC11946904

[13] Shen CJ Chen HC Lin CL Thakur A Onuku R Chen IC . Contribution of prostaglandin E2-induced neuronal excitation to drug resistance in glioblastoma countered by a novel blood–brain barrier crossing celecoxib derivative. Adv Sci. (2025) 12:e06336. doi: 10.1002/advs.202506336. PMID: 40658067 PMC12520548

[14] Chakraborty C Nissen I Remeseiro S . What epigenetics teaches us about neuron-glioma interactions. Bioessays. (2025) 47:e70043. doi: 10.1002/bies.70043. PMID: 40685688 PMC12376016

[15] Venkatesh HS Morishita W Geraghty AC Silverbush D Gillespie SM Arzt M . Electrical and synaptic integration of glioma into neural circuits. Nature. (2019) 573:539–45. doi: 10.1038/s41586-019-1563-y. PMID: 31534222 PMC7038898

[16] Kim KH Migliozzi S Koo H Hong JH Park SM Kim S . Integrated proteogenomic characterization of glioblastoma evolution. Cancer Cell. (2024) 42:358–377.e8. doi: 10.1016/j.ccell.2023.12.015. PMID: 38215747 PMC10939876

[17] Osswald M Jung E Sahm F Solecki G Venkataramani V Blaes J . Brain tumour cells interconnect to a functional and resistant network. Nature. (2015) 528:93–8. doi: 10.1038/nature16071. PMID: 26536111

[18] Weil S Osswald M Solecki G Grosch J Jung E Lemke D . Tumor microtubes convey resistance to surgical lesions and chemotherapy in gliomas. Neuro Oncol. (2017) 19:1316–26. doi: 10.1093/neuonc/nox070. PMID: 28419303 PMC5596180

[19] Sun Y Wang X Zhang DY Zhang Z Bhattarai JP Wang Y . Brain-wide neuronal circuit connectome of human glioblastoma. Nature. (2025) 641:222–31. doi: 10.1038/s41586-025-08634-7. PMID: 39821165 PMC12347542

[20] Koo H Sa JK . Proteogenomic insights into glioblastoma evolution: neuronal reprogramming and therapeutic vulnerabilities. Brain Tumor Res Treat. (2025) 13:81–6. doi: 10.14791/btrt.2025.0018. PMID: 40759475 PMC12329232

[21] Meyer J Yu K Luna-Figueroa E Deneen B Noebels J . Glioblastoma disrupts cortical network activity at multiple spatial and temporal scales. Nat Commun. (2024) 15:4503. doi: 10.1038/s41467-024-48757-5. PMID: 38802334 PMC11130179

[22] Fadel S Ellaithy A . Brain metastasis: incidence, trend analysis, and impact on survival using SEER database (2010–2020). J Clin Oncol. (2025) 43:2021. doi: 10.1200/JCO.2025.43.16_suppl.2021

[23] Savchuk S Gentry KM Wang W Carleton E Biagi-Junior CAO Luthria K . Neuronal activity-dependent mechanisms of small cell lung cancer pathogenesis. Nature. (2025) 646:1232–1242. doi: 10.1038/s41586-025-09492-z. PMID: 40931074 PMC12571889

[24] Zeng Q Michael IP Zhang P Saghafinia S Knott G Jiao W . Synaptic proximity enables NMDAR signalling to promote brain metastasis. Nature. (2019) 573:526–31. doi: 10.1038/s41586-019-1576-6. PMID: 31534217 PMC6837873

[25] Lathia JD Mack SC Mulkearns-Hubert EE Valentim CL Rich JN . Cancer stem cells in glioblastoma. Genes Dev. (2015) 29:1203–17. doi: 10.1101/gad.261982.115. PMID: 26109046 PMC4495393

[26] Yun EJ Kim D Kim S Hsieh JT Baek ST . Targeting Wnt/β-catenin-mediated upregulation of oncogenic Nlgn3 suppresses cancer stem cells in glioblastoma. Cell Death Dis. (2023) 14:423. doi: 10.1038/s41419-023-05967-x. PMID: 37443071 PMC10344874

[27] Venkatesh HS Tam LT Woo PJ Lennon J Nagaraja S Gillespie SM . Targeting neuronal activity-regulated neuroligin-3 dependency in high-grade glioma. Nature. (2017) 549:533–7. doi: 10.1038/nature24014. PMID: 28959975 PMC5891832

[28] Nejo T Krishna S Yamamichi A Lakshmanachetty S Jimenez C Lee KY . Glioma-neuronal circuit remodeling induces regional immunosuppression. Nat Commun. (2025) 16:4770. doi: 10.1038/s41467-025-60074-z. PMID: 40404658 PMC12098748

[29] Groblewska M Litman-Zawadzka A Mroczko B . The role of selected chemokines and their receptors in the development of gliomas. Int J Mol Sci. (2020) 21:3704. doi: 10.3390/ijms21103704. PMID: 32456359 PMC7279280

[30] Yuan W Zhang Q Gu D Lu C Dixit D Gimple RC . Dual role of CXCL8 in maintaining the mesenchymal state of glioblastoma stem cells and M2-like tumor-associated macrophages. Clin Cancer Res. (2023) 29:3779–92. doi: 10.1158/1078-0432.CCR-22-3273. PMID: 37439870

[31] Nilsson CL Dillon R Devakumar A Shi SD Greig M Rogers JC . Quantitative phosphoproteomic analysis of the STAT3/IL-6/HIF1α signaling network: an initial study in GSC11 glioblastoma stem cells. J Proteome Res. (2010) 9:430–43. doi: 10.1021/pr9007927. PMID: 19899826

[32] Lamano JB Lamano JB Li YD DiDomenico JD Choy W Veliceasa D . Glioblastoma-derived IL6 induces immunosuppressive peripheral myeloid cell PD-L1 and promotes tumor growth. Clin Cancer Res. (2019) 25:3643–57. doi: 10.1158/1078-0432.CCR-18-2402. PMID: 30824583 PMC6571046

[33] Wang H Lathia JD Wu Q Wang J Li Z Heddleston JM . Targeting interleukin 6 signaling suppresses glioma stem cell survival and tumor growth. Stem Cells. (2009) 27:2393–404. doi: 10.1002/stem.188. PMID: 19658188 PMC2825688

[34] Lin D Shen L Luo M Zhang K Li J Yang Q . Circulating tumor cells: biology and clinical significance. Signal Transduct Target Ther. (2021) 6:404. doi: 10.1038/s41392-021-00817-8. PMID: 34803167 PMC8606574

[35] Cacho-Díaz B García-Botello DR Wegman-Ostrosky T Reyes-Soto G Ortiz-Sánchez E Herrera-Montalvo LA . Tumor microenvironment differences between primary tumor and brain metastases. J Transl Med. (2020) 18:1. doi: 10.1186/s12967-019-02189-8. PMID: 31900168 PMC6941297

[36] Lowery FJ Yu D . Brain metastasis: unique challenges and open opportunities. Biochim Biophys Acta Rev Cancer. (2017) 1867:49–57. doi: 10.1016/j.bbcan.2016.12.001. PMID: 27939792 PMC5272787

[37] Liu W Powell CA Wang Q . Tumor microenvironment in lung cancer-derived brain metastasis. Chin Med J (Engl). (2022) 135:1781–91. doi: 10.1097/cm9.0000000000002127. PMID: 35838548 PMC9521756

[38] Zhang D Tang DG Rycaj K . Cancer stem cells: regulation programs, immunological properties and immunotherapy. Semin Cancer Biol. (2018) 52:94–106. doi: 10.1016/j.semcancer.2018.05.001. PMID: 29752993 PMC7859848

[39] Kienzler JC Becher B . Immunity in Malignant brain tumors: tumor entities, role of immunotherapy, and specific contribution of myeloid cells to the brain tumor microenvironment. Eur J Immunol. (2024) 54:e2250257. doi: 10.1002/eji.202250257. PMID: 37940552

[40] Walcher L Kistenmacher AK Suo H Kitte R Dluczek S Strauß A . Cancer stem cells–origins and biomarkers: perspectives for targeted personalized therapies. Front Immunol. (2020) 11:1280. doi: 10.3389/fimmu.2020.01280. PMID: 32849491 PMC7426526

[41] Fiori ME Villanova L De Maria R . Cancer stem cells: at the forefront of personalized medicine and immunotherapy. Curr Opin Pharmacol. (2017) 35:1–11. doi: 10.1016/j.coph.2017.04.006. PMID: 28527911

[42] Yang L Shi P Zhao G Xu J Peng W Zhang J . Targeting cancer stem cell pathways for cancer therapy. Signal Transduct Target Ther. (2020) 5:8. doi: 10.1038/s41392-020-0110-5. PMID: 32296030 PMC7005297

[43] Finocchiaro G Pellegatta S . Immunotherapy with dendritic cells loaded with glioblastoma stem cells: from preclinical to clinical studies. Cancer Immunol Immunother. (2016) 65:101–9. doi: 10.1007/s00262-015-1754-9. PMID: 26377689 PMC11029491

[44] Xu Q Liu G Yuan X Xu M Wang H Ji J . Antigen-specific T-cell response from dendritic cell vaccination using cancer stem-like cell-associated antigens. Stem Cells. (2009) 27:1734–40. doi: 10.1002/stem.102. PMID: 19536809 PMC5854496

[45] Phuphanich S Wheeler CJ Rudnick JD Mazer M Wang H Nuño MA . Phase I trial of a multi-epitope-pulsed dendritic cell vaccine for patients with newly diagnosed glioblastoma. Cancer Immunol Immunother. (2013) 62:125–35. doi: 10.1007/s00262-012-1319-0. PMID: 22847020 PMC3541928

[46] Wen PY Reardon DA Armstrong TS Phuphanich S Aiken RD Landolfi JC . A randomized, double-blind, placebo-controlled phase II trial of dendritic cell vaccine ICT-107 in newly diagnosed patients with glioblastoma. Clin Cancer Res. (2019) 25:5799–807. doi: 10.1158/1078-0432.CCR-19-0261. PMID: 31320597 PMC8132111

[47] Hadiloo K Mostanadi P Asadzadeh A Taremi S Esmaeilzadeh A . Targeting cancer stem cells with CAR-based immunotherapy: biology, evidence, and future directions. Cancer Cell Int. (2025) 25:289. doi: 10.1186/s12935-025-03846-3. PMID: 40722155 PMC12302805

[48] Higgins DMO Caliva M Schroeder M Carlson B Upadhyayula PS Milligan BD . Semaphorin 3A mediated brain tumor stem cell proliferation and invasion in EGFRviii mutant gliomas. BMC Cancer. (2020) 20:1213. doi: 10.1186/s12885-020-07694-4. PMID: 33302912 PMC7727139

[49] Andrews DW Judy KD Scott CB Garcia S Harshyne LA Kenyon L . Phase Ib clinical trial of IGV-001 for patients with newly diagnosed glioblastoma. Clin Cancer Res. (2021) 27:1912–22. doi: 10.1158/1078-0432.CCR-20-3805. PMID: 33500356

[50] Cultrara C Uhl C Kirby K Abed Elrazaq E Zellander A Andrews DW . A biologic-device combination product delivering tumor-derived antigens elicits immunogenic cell death-associated immune responses against glioblastoma. J Immunother Cancer. (2023) 11:e006880. doi: 10.1136/jitc-2023-006880. PMID: 37550054 PMC10407365

[51] Zilberberg J Uhl C Scott CB Andrews DW Exley MA . Broad applicability of the Goldspire™ platform for the treatment of solid tumors. Clin Immunol. (2024) 268:110373. doi: 10.1016/j.clim.2024.110373. PMID: 39349152

[52] Exley MA Garcia S Zellander A Zilberberg J Andrews DW . Challenges and opportunities for immunotherapeutic intervention against myeloid immunosuppression in glioblastoma. J Clin Med. (2022) 11:1069. doi: 10.3390/jcm11041069. PMID: 35207340 PMC8880446

